# Fibre à myéline: diagnostic différentiel de l’œdème papillaire

**DOI:** 10.11604/pamj.2016.25.33.5523

**Published:** 2016-09-27

**Authors:** Aniss Regragui, Rajae Daoudi

**Affiliations:** 1 Université Mohammed V Souissi, Service d’Ophtalmologie A de l’Hôpital des Spécialités, Centre Hospitalier Universitaire, Rabat, Maroc

**Keywords:** Fibre à myéline, oedème papillaire, myopie, Fiber myelin, papilledema, myopia

## Image en médecine

La patiente présente dans ce cas est âgée de 32 ans, sans antécédents particuliers et suivie pour myopie depuis l'âge scolaire, lors d'un examen de routine; on trouve au niveau des 2 yeux une acuité visuelle a 10/10 une cornée claire, une chambre antérieure, de bonne profondeur, un iris de trame et coloration normale et un cristallin clair avec un tonus oculaire a 16 mmhg à l'oeil droit et 14 mmhg à l'oeil gauche. L'examen du fond d'oeil trouve au niveau de l'oeil gauche une papille d'excavation 3/10 avec une tache blanchâtre mal limitée à bord flou contiguë au bord nasal de la papille évoquant l'aspect de fibre a myéline, l'oeiladélphe est sans particularités. L'angiographie à la fluoresceïne rétinienne ne montre ni retard de remplissage ni des signes de fuite précoce ou tardif éliminant l'œdème papillaire. Les fibres à myéline c'est une pathologie bénigne asymptomatique de découverte fortuite lors d'un examen de fond d'oeil et qui peut être associé a un vice réfractif le plus souvent la myopie et réalisant un diagnostic différentiel devant l'aspect d'œdème papillaire.

**Figure 1:**
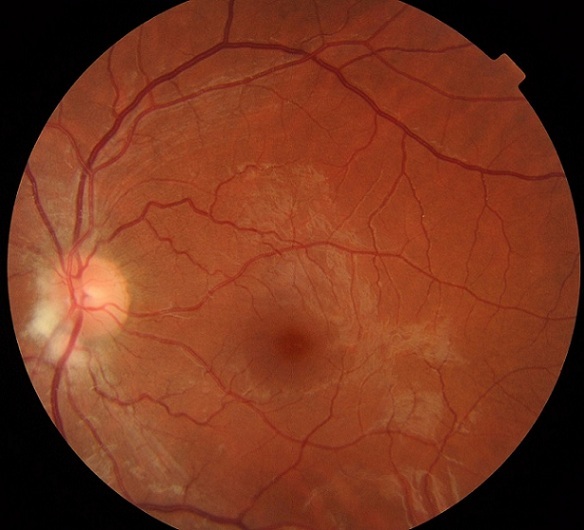
Tâche blanchâtre mal limitée à bord flou contiguë au bord nasal de la papille évoquant l´aspect de fibre a myéline

